# *Borrelia persica* infection in wild carnivores in Israel: molecular characterization and new potential reservoirs

**DOI:** 10.1186/s13071-023-05953-4

**Published:** 2023-09-26

**Authors:** Dor Shwartz, Yaarit Nachum-Biala, Stephanie Oren, Kobi Aharoni, Nir Edery, Lior Moss, Roni King, Roi Lapid, Reinhard K. Straubinger, Gad Baneth

**Affiliations:** 1 Koret School of Veterinary Medicine, Hebrew University of Jerusalem, P.O. Box 12, 7610001 Rehovot, Israel; 2 Pathology Department, Kimron Veterinary Institute, Rishon Lezion, Israel; 3 Israeli National Parks and Nature Reserves, Jerusalem, Israel; 4 Bacteriology and Mycology, Institute for Infectious Diseases and Zoonoses, Ludwig-Maximilians-Universität München, Oberschleißheim, Germany

**Keywords:** *Borrelia persica*, Borreliosis, European badger, Relapsing fever, Striped hyena, Wildlife carnivores

## Abstract

**Background:**

*Borrelia persica* causes tick-borne relapsing fever in Israel, the eastern Mediterranean basin, and Asia. Relapsing fever is associated with severe illness and potentially death in humans and animals. Since *B. persica* infection has rarely been described in wild animals, the aim of this study was to evaluate the prevalence of infection with *B. persica* in wild carnivores in Israel.

**Methods:**

Spleen and blood clot samples from wild carnivores, which underwent necropsy, were tested for the presence of *Borrelia* DNA by real-time polymerase chain reaction (PCR). PCR products were sequenced, and the spirochete loads were quantified using a specific quantitative PCR (qPCR).

**Results:**

A total of 140 samples from 74 wild carnivores were analyzed for the presence of *Borrelia* DNA. Six out of the 74 (8.1%) animals were found positive for *B. persica* by PCR and sequencing of the flagellin B gene, of which 4/74 (5.4%) were also positive by PCR for the glycerophosphodiester phosphodiesterase (*glpQ*) gene. Positive samples were obtained from three European badgers, and one striped hyena, golden jackal, and red fox each. All *B. persica*-positive animals were young males (*P* < 0.0001). Quantifiable results were obtained from 3/5 spleen and 4/5 blood samples. The spirochete loads in the blood were significantly higher than those found in the spleen (*P* = 0.034).

**Conclusions:**

The prevalence of *B. persica* infection found in wild carnivores brought for necropsy was unexpectedly high, suggesting that this infection is widespread in some wild animal species in Israel. This is the first report of *B. persica* infection in the European badger and striped hyena. These carnivores have a wide geographical range of activity, and the results of this survey raise the possibility that they may serve as reservoir hosts for *B. persica*.

**Graphical Abstract:**

**Figure:**
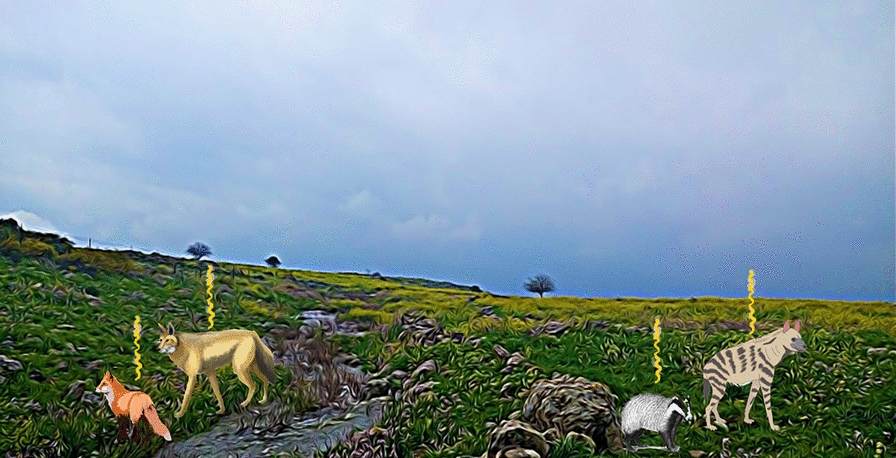

## Background

Relapsing fever is a zoonotic infectious disease caused by spirochetes from the genus *Borrelia*. The disease in humans is characterized by recurrent fever episodes, headaches, lethargy, tachycardia, conjunctivitis, hepatomegaly, splenomegaly, pigmenturia, vomiting, myalgia, and arthralgia [1, 2]. Relapsing fever can be divided into the louse-borne relapsing fever, caused by *Borrelia recurrentis* and transmitted by the human body louse *Pediculus humanus*, and tick-borne relapsing fever (TBRF), which is mostly transmitted by argasid ticks from the genus *Ornithodoros* [3, 4]. The TBRF agents include also a small group of species, which are transmitted by ixodid ticks, such as *Borrelia miyamotoi* transmitted by ticks of the genus* Ixodes*, *Borrelia theileri* transmitted by *Rhipicephalus* spp., and *Borrelia lonestari* transmitted by *Amblyomma americanum* [5].

Relapsing fever caused by *Borrelia persica* has been reported in humans from Iran, Pakistan, several Central Asian countries, and Egypt, and it is the only reported endemic relapsing fever species in Israel [6–9]. Mortality due to *B. persica* infection in humans is low in patients treated with antibiotics and monitored during therapy; however, fatal infections with severe complications have been reported in Israel [10, 11]. *Ornithodoros tholozani*, the vector of *B. persica*, is common in Israel and is among the few soft tick species found in the country [12]. The geographical distribution of *O. tholozani* extends from India and Central Asia to Egypt and overlaps the distribution of *B. persica* [13, 14]. Like other *Ornithodoros* spp., *O. tholozani* ticks are rapid blood feeders, with all life stages feeding on warm-blooded mammals, including humans, rodents, cattle, small ruminants, camels, foxes, jackals, porcupines, and hedgehogs [4, 13]. *Ornithodoros tholozani* is commonly found in shaded locations, including caves, ruins, and rock crevices [14]. The reported incidence of TBRF in civilians in Israel has declined from 0.35 cases per 100,000 inhabitants in the years 1975 to 1985, to 0.11/100,000 from 1986 to 2003. Nevertheless, the incidence among Israeli military personnel remained stably high with an annual average of 6.4 cases per 100,000 [15].

Rodents are considered the main reservoir hosts for the North American TBRF species *Borrelia hermsii*, *Borrelia parkeri*, and *Borrelia turicatae*, as well as for the Old World species *Borrelia hispanica* and *Borrelia crocidurae* [16–19]. Infection with *B. persica* in animals in Israel has been described in domestic dogs and cats, in which the infection is associated with clinical disease and clinicopathological findings such as fever, lethargy, anorexia, anemia, and thrombocytopenia, and can be fatal [20, 21]. Clinical manifestations of relapsing fever were also observed in dogs infected with the North American species *B. turicatae* and *B. hermsii*, and in cats infected with *B. hispanica* [22–24]. Previously, *B. persica* was identified by molecular methods in several wildlife species in Israel, mostly herbivores and insectivores [25, 26].

According to the World Health Organization (WHO) reports, zoonotic diseases are responsible for about 60% of all emerging infectious diseases (EID), with an estimated global morbidity of 10^7^ patients and several million human fatalities annually [27]. Wild animals were found to play a major role in the global burden caused by EID, with more than 70% of zoonotic EID originating in wildlife [28]. Wild carnivores are an important part of the sylvatic cycle in different zoonotic diseases, such as rabies, cystic echinococcosis, and trichinellosis [29–31]. The close proximity between wild animals and humans, even in urban areas, may contribute to the role of the former in human infection [32]. Therefore, knowledge of the prevalence of *B. persica* infection in wild carnivores in Israel and their possible part in the epidemiology of relapsing fever is essential. The aims of this study were to survey a variety of wild carnivore species in Israel for infection with *B. persica* and to evaluate the bacterial loads in infected animals. Sampling of Indian crested porcupines (*Hystrix indica*), which are large omnivore rodents that prey on small vertebrates [33], was also included in the study, because they were identified in a previous study as the main source for blood meal in *O. tholozani* ticks surveyed in Israel [25].

## Methods

### Sample collection and DNA extraction

Spleen and blood samples were collected from wild carnivores that underwent post-mortem examination at the Pathology Department of the Kimron Veterinary Institute in Beit Dagan, Israel. Data collected included the carcass's geographical point of collection, species, sex, presumptive age, and necropsy report. There were no data regarding the animal’s clinical signs prior to death. Samples were preserved at −80 °C until DNA extraction was conducted. DNA was extracted from 10 mg of spleen and 100 µl blood using a commercial kit (DNeasy Blood & Tissue Kit, Qiagen, Germany) following the manufacturer's protocol, with some modifications; spleen samples were washed with phosphate-buffered saline (PBS) and mechanically crushed before the standard protocol was performed.

### Molecular identification and genetic characterization of *Borrelia* spp.

Screening of samples for *Borrelia* DNA was performed by real-time quantitative polymerase chain reaction (qPCR) using primers Bfpbu (5′-GCTGAAGAGCTTGGAATGCAACC-3′) and Bfpcr (5′-TGATCAGTTATCATTCTAATAGCA-3′) targeting a 346-base-pair (bp) fragment of the flagellin (*flaB*) gene of *Borrelia* [34]. Positive samples were also tested for the relapsing fever-specific gene glycerophosphodiester phosphodiesterase (*glpQ*) by real-time PCR using primers 510f (5′-AAAACCCTTTTGGCATAAACAACA-3′) and 770R (5′-CCAGGGTCCAATTCCGTCAG-3′) targeting a 280-bp fragment of the gene [35]. The *flaB* and *glpQ* real-time PCR protocols were carried out with an initial hold for 3 min at 95 °C, followed by 45 cycles of 15 s at 95 °C, 30 s at 58 °C, and 10 s at 72 °C. The melting phase started at 60 °C, each step rising by 0.3 °C, and finished at 95 °C with a hold for 90 s at the first step and 5 s at the subsequent steps. Each reaction was performed in 20 μl reaction volume containing 4 μl of DNA, 0.5 μM of each primer, 0.6 μl of syto9 (Invitrogen, CA, USA.), 4.4 μl of distilled extra-pure water, and 10 μl of DreamTaq PCR Master Mix (Thermo Scientific, Loughborough, UK). DNA extracted from *B. persica* culture grown in the authors' laboratory was used as a positive control. All reactions were performed in the Life Technologies StepOnePlus Real-Time PCR System (Thermo Fisher Scientific, Waltham, MA, USA). Samples were considered positive when the melting temperature (T_m_) was equal to the positive control T_m_ and found to be 82 °C for the *flaB* gene and 77.6 °C for the *glpQ* gene. Positive PCR products were sequenced from both directions using the respective forward and reverse primers. No purification was made prior to sequencing. Sequencing was performed by the BigDye Terminator cycle sequencing chemistry from Applied Biosystems ABI 3700 DNA Analyzer (ABI, Carlsbad, CA, USA) and the ABI Data collection and Sequence Analysis software at the Center for Genomic Technologies, Hebrew University of Jerusalem, Israel. DNA sequences were aligned using the Molecular Evolutionary Genetics Analysis (MEGA) software, version 11.013 [36]. Sequences were further compared with the GenBank database using the BLAST algorithm (National Center for Biotechnology Information, Bethesda, MD, USA; http://blast.ncbi.nlm.nih.gov/Blast.cgi). Species-level identification was obtained according to the closest BLAST match when sequences showed identity higher than 99% to GenBank sequences.

### Quantification of *B. persica* in spleen and blood samples

Quantitative PCR was performed on the positive spleen and blood samples by amplifying a 346-bp fragment of the *flaB* gene of *Borrelia* [34]. The purpose of quantifying the *B. persica* bacterial load was to validate infection by characterizing the numbers of spirochetes in the blood and spleen, and to compare the bacterial load in the different tissues.

Standard curves for spleen and blood were generated using 10-fold dilutions of *B. persica* grown in culture, from an isolate originally cultured from a cat in Israel and designated as the *B. persica* LMU-C01 strain [37]. Cultured spirochetes were counted by dark-field microscopy using a Neubauer chamber cell (Neubauer improved, Boeco, Germany). Ten milligrams of spleen and 100 µl of blood from a golden jackal which tested negative for the presence of *Borrelia* spp. by PCR were spiked with 6 × 10^7^ cultured *B. persica* organisms. DNA was extracted from the spiked spleen and blood using the DNeasy Blood & Tissue Kit (Qiagen, Germany). The extracted DNA was diluted with ultra-pure DNA-free water to reach the concentrations of 6 × 10^7^ to 6 × 10^1^ spirochetes per sample. Standard curves were obtained by running duplicates of the spiked spleen and blood with culture dilution reference points and plotting their cycle threshold (Ct) values against the log of *B. persica* absolute numbers using the StepOne software version 2.2.2 (Applied Biosystems, Thermo Fisher Scientific, Foster City, CA, USA). Real-time PCR and reaction mix protocols were carried out as described for the *flaB* gene.

In order to correct *B. persica* quantities to host DNA quantity in the spleen and blood samples, a real-time PCR was performed to detect mammalian DNA by targeting a 150-bp fragment of vertebrate *18S rRNA* gene using universal primers 0033F (5′-TTCTAGAGCTAATACATGCCGA-3′) and 0049R (5′-CGAGGTTATCTAGAGTCACC-3′) specific for vertebrates as previously described [38]. Real-time PCR, the melting phase, and the components of the reaction were the same as for the amplification of the *flaB* and *glpQ* genes. DNA extracted from a culture of *B. persica* was used as a negative control. To obtain the actual loads of spirochetes, *B. persica flaB* Ct values were adjusted by calculating the difference between the actual mammal *18S rRNA* gene Ct obtained for each sample and the average mammal *18S rRNA* gene Ct values, and adding this value to the *Borrelia flaB* Ct value for each sample. Finally, the *B. persica* load (absolute number per millimeter of blood and milligram of spleen) in each sample was calculated according to the linear regression equation of the standard curve (Cт = m [log (Qty)] + b; where m is the slope, b is the y-intercept, and Qty is the standard quantity), which was obtained using StepOne version 2.2.2 software (Thermo Fisher Scientific).

### Statistical analysis

Statistical analysis was performed using the SPSS software version 26.0 (SPSS IBM, Armonk, NY, USA). Comparisons of sex and age differences between positive and negative animals were tested using the Chi-square test. The continuity correction was employed when 2 × 2 tables were used. Bacterial loads in blood samples as compared to spleen samples were tested by the non-parametric Mann–Whitney test for two independent variables. A Pearson correlation coefficient was computed to assess the linear relationship between spirochetal loads in the spleen and in the blood. Statistical significance was defined as *P* < 0.05. Maps showing the collection sites were constructed using the ArcGIS Map 10.0 software (Esri, Redlands, CA, USA).

## Results

A total of 140 samples (68 blood and 72 spleen samples) from 74 wild carnivores, obtained by the Pathology Department at the Kimron Veterinary Institute from November 2020 to September 2022, were analyzed for the presence of *B. persica* DNA. The specimens originated from wild animals of five families and eight species, including the golden jackal (*Canis aureus*), red fox (*Vulpes vulpes*), wolf (*Canis lupus*), Egyptian mongoose (*Herpestes ichneumon*), striped hyena (*Hyaena hyaena*), Indian crested porcupine (*Hystrix indica*), European badger (*Meles meles*), and beech marten (*Martes foina*) (Table 1). The most frequent cause of admission for post-mortem examination was culling wild carnivores as part of a rabies monitoring programs (79.7%), followed by road kills (19.0%) and predation (1.3%).

**Table 1:** Wild animal species sampled and rate of infection with *Borrelia persica* in blood and spleen samples

Family	Species	Species scientific name	No. of animals tested	No. of *B. persica*-infected animals	% of *B. persica*-infected animals	Positive in blood sample	Positive in spleen sample	Location of positive animals
Canidae	Golden jackal	*Canis aureus*	40	1	2.5	0/1	1/1	Tel Aviv
	Red fox	*Vulpes vulpes*	8	1	12.5	1/1	1/1	Ein Zivan
	Wolf	*Canis lupus*	1	0				
Herpestidae	Egyptian mongoose	*Herpestes ichneumon*	1	0				
Hyaenidae	Striped hyena	*Hyaena hyaena*	3	1	33.3	1/1	0/1	Motza Illit
Hystricidae	Indian crested porcupine	*Hystrix indica*	3	0				
Mustelidae	European badger	*Meles meles*	16	3	18.7	3/3	3/3	Shlomi, Aminadav, Northern Israel
	Beech marten	*Martes foina*	2	0				
Total			74	6	8.1	5/6	5/6	

Out of 74 wild animals, six (8.1%) were found positive for the presence of *Borrelia* spp. DNA by PCR amplification and sequencing of the *flaB* gene. All positive samples showed > 99% identity to *B. persica* GenBank accession number KY964442.1 previously amplified from a rock hyrax in Israel. All samples were also tested by the *glpQ* PCR, and four (5.4%) were found positive and showed > 99% identity to *B. persica* GenBank accession number MF401448, which was also amplified from a rock hyrax in Israel. All four animals positive for the *glpQ* PCR were also positive for the *flaB* PCR. All PCR-positive animals had both spleen and blood samples available. Four out of six (67%) positive animals were positive in both spleen and blood, 1/6 (16.7%) was positive only in the spleen, and 1/6 (16.7%) was positive only in the blood.

Positive samples (Table 1) were obtained from three European badgers (*M. meles*), one striped hyena (*H. hyaena*), one golden jackal (*C. aureus*), and one red fox (*V. vulpes*). Overall, one of three (33.3%) striped hyenas was found positive, followed by the European badgers (3/16, 18.75%), red fox (1/8, 12.5%), and golden jackal (1/40, 2.5%).

No significant differences were found in the prevalence between the positive animals (*χ*^2^ = 6.237, *df* = 3, *P* = 0.101).

Sex and age were known for 54 and 38 animals, respectively. Twenty-eight out of 54 (51.9%) animals for which the sex was documented were females, and 26/54 (48.1%) were males. Furthermore, 29/38 (76.3%) animals, for which the age was documented, were adults, whereas 9/38 (23.7%) were juvenile. All positive animals were young-age males (Table 2). Sex and age proportions in the *B. persica*-positive animals were found significantly different from those proportions in the general study population (*χ*^2^ = 5.120, *df* = 1. *P* = 0.024; *χ*^2^ = 18.218, *df* = 1, *P* < 0.0001, respectively). Eleven out of 74 animals tested were found infested with ixodid ticks.

**Table 2:** Sex and age details available in the study population and the *Borrelia persica*-positive animals

	General study population		*B. persica-*positive animals		*P*-value
Sex	Female	Male	Female	Male	
	28/54	26/54	0/6	6/6	0.011
Age	Young	Adult	Young	Adult	
	9/38	29/38	6/6	0/6	0.033

**Fig. 1:**
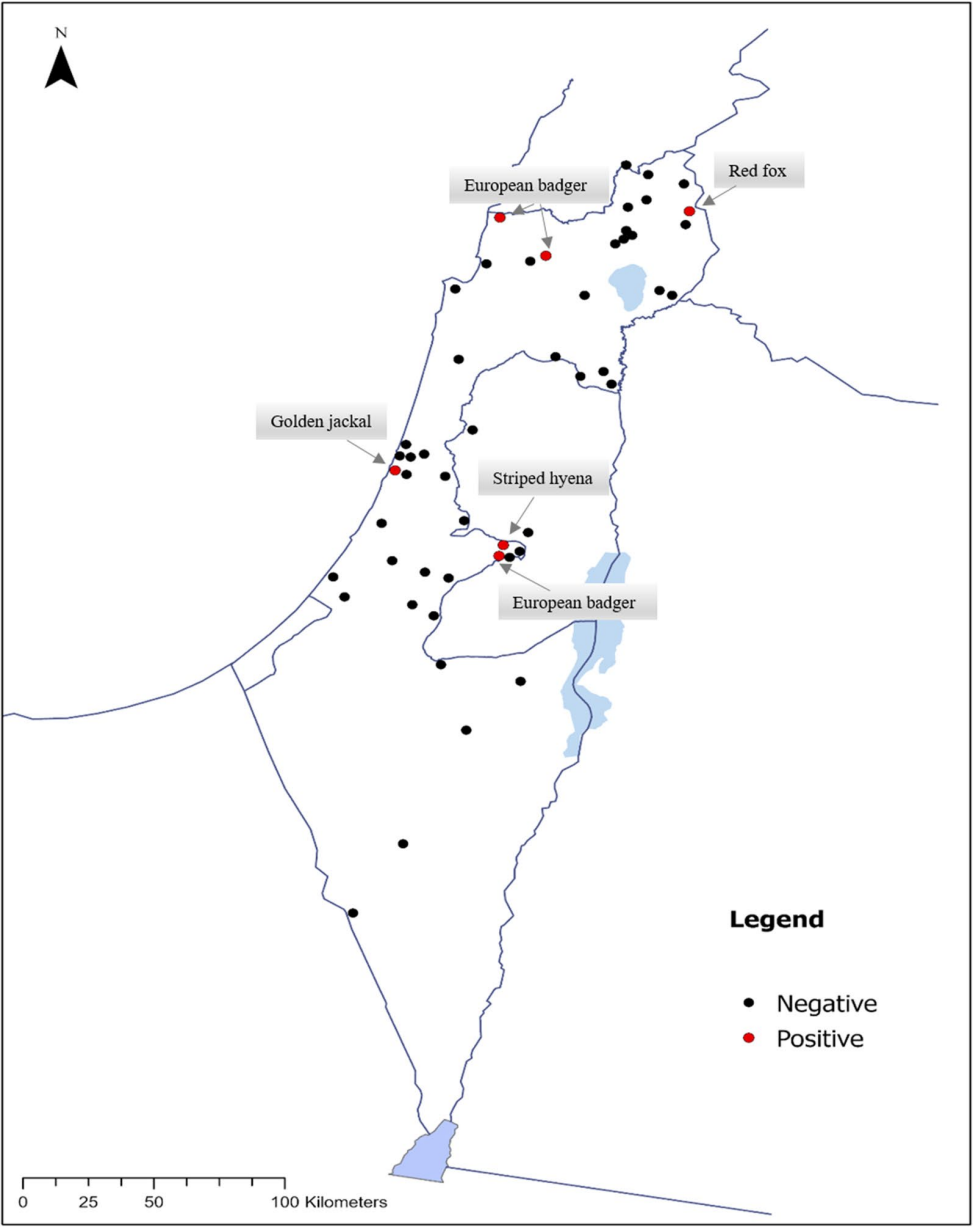
Geographical distribution of sampled wild carnivores indicating the presence or absence of infection with *B. persica*. The locations of positive animals are marked in red dots, and locations where negative animals were found are marked in black dots. Positive animals are highlighted with its species name

The geographical distribution of the animals tested ranged from Metula and Ein Zivan in northern Israel to Mount Sagi in the south of the country (Fig. 1). Positive animals were found in six out of 51 distinct geographical locations, including three animals from northern Israel and three from the central part of the country (Fig. 1, Table 1).

Quantifiable numbers of *B. persica* were available from 3/5 (60%) PCR-positive spleen samples and 4/5 (80%) blood samples (Table 3). The minimal detection limit was determined by the concentration for which the assay maintained linearity for spleen and for blood samples and was found to be as low as 63 spirochetes per 1 mg of spleen and 6333 spirochetes per 1 ml of blood. The minimal detection limit was equal for the spleen and the blood as the concentrations for these two tissues were per 10 mg for the spleen and per 1 ml for the blood tissue. After normalization with mammalian DNA, the median *B. persica* load in the three quantifiable spleen samples was 2903 spirochetes/mg spleen (range 244–12,673 spirochetes/mg), and for the four quantifiable blood samples, it was 257,086 spirochetes/ml blood (range 14,514–1,547,764 spirochetes/ml). Two spleen and one blood samples harbored a small quantity of *B. persica* DNA that was below the quantifiable detection limit of the assay (Table 3). The absolute numbers of *B. persica* spirochetes based on the *flaB* amplification were significantly higher in the blood compared to the spleen (*U*_(7)_ < 0.0001, *Z* = −2.121, *P* = 0.034). In addition, an almost significant correlation was found between spleen and blood spirochete loads of the same animals (*R*^2^ = 0.997*, P* = 0.051).

**Table 3:** *Borrelia persica* loads in blood and spleen samples

Animal number	Blood (per ml)	Spleen (per mg)
399950	14,514	244
419856	457,419	2903
421617	1,547,764	12,673
450595	56,753	ND
451407	ND	ND

ND: samples in which quantification was not possible

**Fig. 2:**
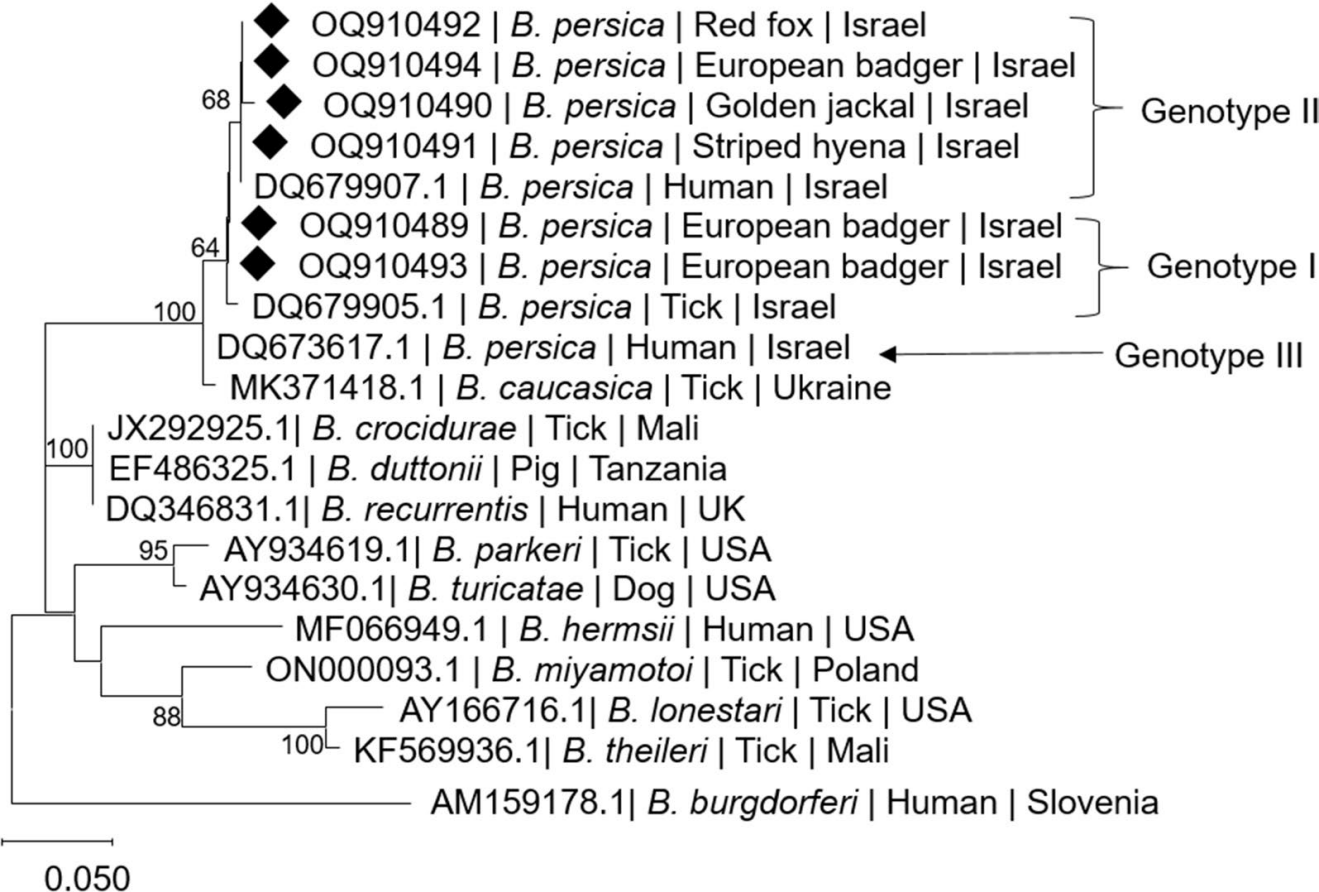
A maximum likelihood phylogram comparing 229-bp DNA fragment sequences of the *flaB* gene from the wild carnivores included in the study to sequences from other *B. persica* and other *Borrelia* spp. GenBank accessions. New sequences derived from this study are marked with black diamond squares. Note the division into *B. persica* genotypes marked in Roman numerals. The GenBank accession numbers, species of infected host, and country of origin are included for each sequence. The Tamura 3-parameter model was used in the construction of this phylogram with bootstrap performed on 1000 replicates, and values higher than 60% are indicated

Phylogenetic analysis based on a 229-bp segment of the *flaB* gene sequence (Fig. 2) revealed that all *B. persica* sequences from this study clustered together. Four sequences of the *B. persica-*positive animals clustered together with a *B. persica* genotype II sequence, previously amplified from a human patient in Israel (GenBank DQ679907.1), whereas two sequences from positive animals clustered together with a *B. persica* genotype I sequence from an *O. tholozani* tick collected in Israel (GenBank DQ679905.1) [39]. None of the sequences from the wildlife study clustered with the *B. persica* genotype III sequence, which originated from a human patient in Israel (GenBank DQ673617). All *B. persica* sequences clustered separately from other Old World relapsing fever *Borrelia* spp. including *B. recurrentis*, *B. duttonii*, and *B. crocidurae*, except for *Borrelia caucasica*, which clustered together with *B. persica* genotype III sequence. North American relapsing fever species including *B. parkeri* and *B. turicatae* also clustered separately. TBRF species which are transmitted by ixodid hard ticks including *B. lonestari* and *B. theileri* clustered together, but separately from *B. miyamotoi*, which clustered together with *B. hermsii*.

**Fig. 3:**
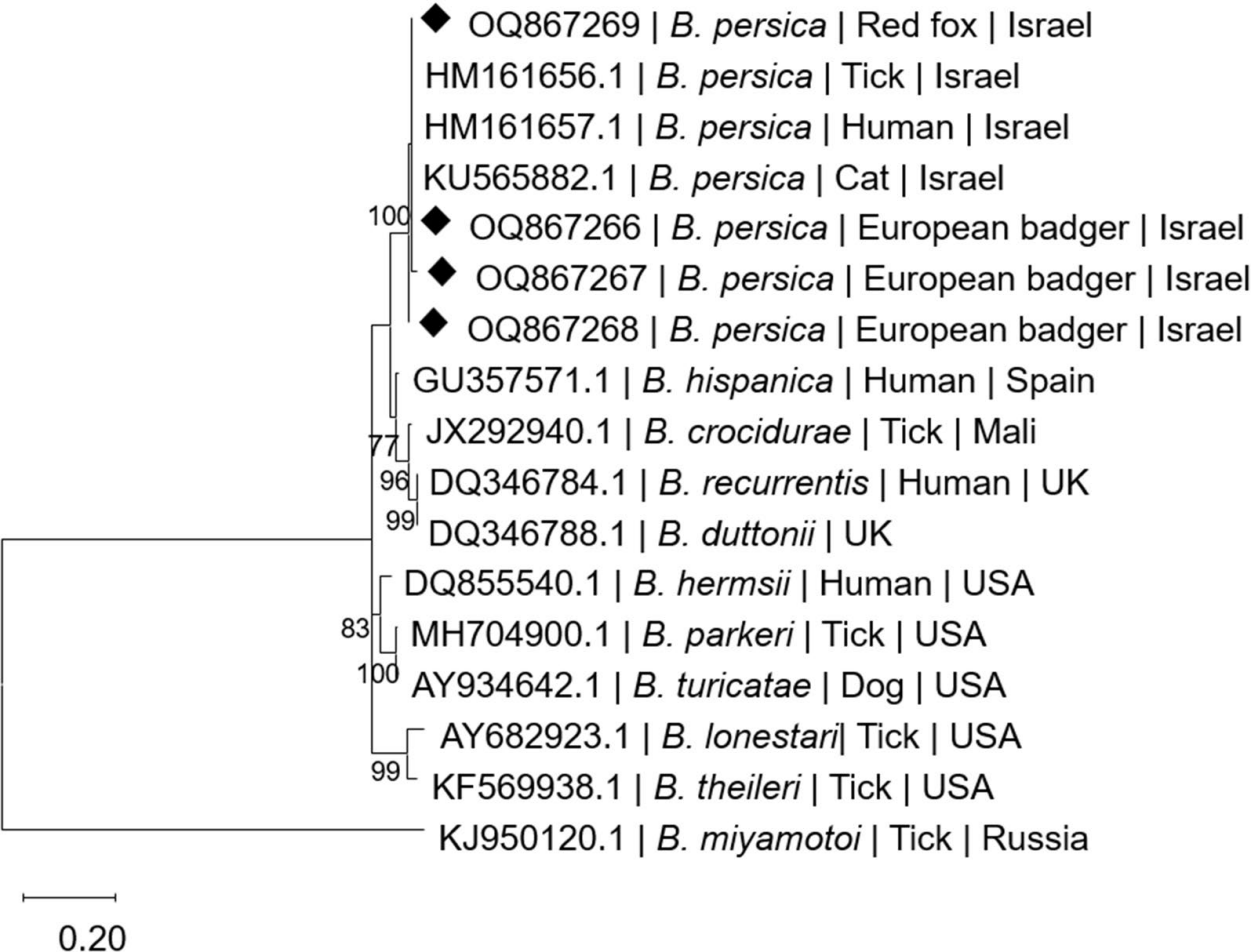
A maximum likelihood phylogram comparing 260-bp DNA fragment sequences of the *glpQ* gene from the wild carnivores included in the study to sequences from other *B. persica* and other *Borrelia* spp. GenBank accessions. New sequences derived from this study are marked with black diamond squares. The GenBank accession numbers, species of infected host, and country of origin are included for each sequence. The Tamura-3-Parameter model was used in the construction of this phylogram with bootstrap performed on 1000 replicates, and values higher than 70% are indicated

Phylogenetic analysis based on a 260-bp segment of the *glpQ* gene (Fig. 3) also revealed that sequences from *B. persica*-positive wild animals clustered with each other and with other *B. persica* sequences from a human, cat, and an *O. tholozani* tick. As in the *flaB* phylogram, *B. persica glpQ* sequences clustered separately from Old World relapsing fever *Borrelia* spp. including *B. hispanica*, *B. duttonii*, *B. crocidurae*, and *B. recurrentis*. The Old World species including *B. persica* also clustered separately from American relapsing fever *Borrelia* spp., namely *B. hermsii*, *B. parkeri*, and *B. turicatae*.

## Discussion

This study describes infection with the TBRF spirochete *B. persica* in 8% of wild carnivores examined in Israel, and includes the first report of this infection in the European badger and striped hyena. Infection with *B. persica* accompanied by clinical disease was previously described in humans, dogs, and cats [15, 20]. A previous study from Israel, in which samples from domestic dogs and cats admitted to a veterinary teaching hospital for treatment unrelated to relapsing fever were tested using a PCR-based protocol, found that the prevalence of *B. persica* was 1.9% in the dog group and 2.9% in the cat group [21]. Other studies pertaining to animals from Israel reported that *B. persica* infection was detected by PCR in 7% of the screened wild animals, including the social vole (*Microtus socialis*), fat sand rat (*Psammomys obesus*), Cairo spiny mouse (*Acomys cahirinus*), red fox (*V. vulpes*), and golden jackal (*C. aureus*), and in 8% of tested rock hyraxes (*Procavia capensis*) in a second study [25, 26]. Clinical signs of disease have not been described in these previous reports of infection in wildlife in Israel. The infected animals in the current study belong to three different carnivoran families and include the golden jackal, red fox, and the newly reported European badger and striped hyena.

Wild animals are considered reservoirs for several viral, bacterial, protozoal, and helminthic zoonoses [40, 41]. Over the past decade, 51.1% of the reported cases of rabies in Israel were diagnosed in wild carnivores, including golden jackals, red foxes, wolves, and European badgers [42]. The European badger is widely distributed across Europe, the Middle East, and several countries in Western Asia, and it is known to serve as a reservoir host for zoonotic pathogens, such as the tuberculosis bacterium *Mycobacterium bovis*, the Lyme borreliosis spirochete *Borrelia afzelii*, and species of the nematode *Trichinella* [41, 43–45]. A previous study from Israel demonstrated that 72 out of 2777 (2.6%) *O. tholozani* ticks collected in 16 caves were found positive for *B. persica* DNA by PCR. Out of 72 infected ticks, 50 were positive for the presence of host blood. Two ticks (4%) were found positive for *B. persica* infection with host blood from the European badger [25]. To the best of our knowledge, our findings represent the first published data regarding infection of the European badger with any relapsing fever *Borrelia* spp. These findings in which three of 16 badgers were naturally infected with *B. persica* and the fact that *O. tholozani* ticks with badger blood were found to be infected with *B. persica* in a previous study strengthen the suggestion that the European badger may serve as a natural reservoir host for *B. persica* transmission.

The striped hyena is a solitary predator distributed over Northern and Eastern Africa, the Middle East, and India [46]. Its individual range of activity is very wide and documented to reach 72 and 44 km^2^ for males and females, respectively [47]. In Israel, an increase in hyena population density has been observed over the past few decades, with about two individuals per 100 km^2^ compared to 0.06 individuals per 100 km^2^ in the 1970s [48]. In contrast to the European badger, hyenas are less common reservoirs for human infections, with only a few reports of zoonotic pathogen infection in wild hyenas [49, 50]. This is the first report of infection with *Borrelia* spp. in the striped hyena. In a previous study, three *O. tholozani* nymphs were found positive for the presence of host blood from striped hyenas by PCR [25]. These findings support the possibility that the striped hyena may serve as a reservoir host for *B. persica* infection as well. The striped hyena is also capable of distributing *B. persica* between distant locations due to its wide range of activity.

The European badger and the striped hyena both have a long lifespan of about 8–15 years [51, 52], and when their activity ranges and geographical distributions are taken into account, it is suggested that these newly found hosts for *B. persica* could serve as potential reservoirs for transmission of the pathogen to humans and animals via ticks.

The geographical distribution of samples collected during this study covers large areas of Israel and overlaps much of the distribution area of *O. tholozani*, which is found throughout Israel except for the southern part of the Negev desert in southern Israel [14]. This is supported by a previous study on the prevalence of TBRF in Israel which demonstrated a relationship between this geographical area and the presence of clinical disease cases in humans [53]. *Borrelia persica*-positive wild carnivores were also distributed widely across Israel in the current study, with representation for the Jerusalem, Tel Aviv, the Western Galilee, and the Golan Heights areas in central and northern Israel.

Age and sex were found significant factors for infection with *B. persica* in our study, with only young males found infected with *B. persica* [20, 26]. In a previous study that compared the prevalence of TBRF in children and adult humans, the average age among TBRF patients was 21.3 years old with 33% of the patients under 18 years old. Furthermore, out of 92 patients, 82% were males [54]. A likely explanation for young animals being more susceptible to *B. persica* infection is the possible development of resistance in older animals after initial infection at a young age, incomplete maturity of the immune system, or immune suppression due to co-infection, with reduced capability of successful defense against this infection. Infectious agents, such as the canine distemper virus, are associated with immunodeficiency in young animals and were described in the European badger, red fox, and golden jackal [55–57].

Both spleen and blood samples were found suitable for the molecular detection of *B. persica* infection. This is similar to findings from a previous study in hyraxes where the spirochete burden in the blood was up to 1000 times that in the spleen [26]. In the previous study on the prevalence of *B. persica* in rock hyraxes, spirochete loads were quantified by the measurement of the *16S rRNA* gene copies and ranged from 2.3 × 10^4^ to 1 × 10^6^ copies in the spleen and from 5 × 10^6^ to 9.2 × 10^8^ in the blood [26]. Nevertheless, this is the first study to report an absolute quantification of *B. persica* in tissues using an in vitro cultivated culture. Absolute *B. persica* quantities in the spleen and blood were measured using calibration curves of spiked spleen and blood with a known quantity of spirochetes. The spiking method was used in order to balance the effect of internal inhibitors in the different tissues. The spiking process was done using the same culture and DNA extraction method, and therefore the results accurately represent the absolute quantity of *B. persica* spirochetes in the infected tissues.

Wild carnivore populations are expanding rapidly in many parts of Israel and encroaching into human habitation [48, 58]. The potential role of wild carnivores as reservoirs of *B. persica* and their proximity to human settlements may be considered as a risk factor for human and companion animal infection, which control programs for TBRF should address. In contrast to the limited data regarding TBRF in wild carnivores, the occurrence of Lyme borreliosis in those animals was more comprehensively studied, and evidence of infection with *Borrelia burgdorferi*, *B. afzelii*, and *Borrelia garinii* was demonstrated in several wild carnivore species, including the raccoon (*Procyon lotor*)*,* red fox*,* raccoon dog (*Nyctereutes procyonoides*)*,* European badger, pine marten (*Martes martes*), and stone marten (*M. foina*) [59]. Control and management programs aim to reduce infection rates of various infectious agents, including Lyme borreliosis, in wild carnivores through oral vaccination programs. In the case of Lyme borreliosis, studies in experimentally infected laboratory mice have shown protection via oral-route vaccination with live recombinant *Escherichia coli* or vaccinia virus expressing the OspA protein of *B. burgdorferi* [60]. This strategy may also be beneficial for protection against *B. persica* infection in wild carnivores.

The present study had several limitations, which include a relatively limited number of wild carnivores surveyed, a small representation of individual animal species, and missing information on some of the animals. Despite these limitations, the study revealed important information on the high prevalence of *B. persica* infection in wild carnivores in Israel and demonstrated that this infection is widely spread in the surveyed areas.

## Data Availability

All data generated or analyzed during this study are included in this published article.
